# Voluntary movement affects simultaneous perception of auditory and tactile stimuli presented to a non-moving body part

**DOI:** 10.1038/srep33336

**Published:** 2016-09-13

**Authors:** Qiao Hao, Hiroki Ora, Ken-ichiro Ogawa, Taiki Ogata, Yoshihiro Miyake

**Affiliations:** 1 Department of Computational Intelligence and Systems Science, Tokyo Institute of Technology, Yokohama, Japan; 2 Department of Computer Science, Tokyo Institute of Technology, Yokohama, Japan; 3 Research into Artifacts, Center for Engineering (RACE), the University of Tokyo, Kashiwa, Japan

## Abstract

The simultaneous perception of multimodal sensory information has a crucial role for effective reactions to the external environment. Voluntary movements are known to occasionally affect simultaneous perception of auditory and tactile stimuli presented to the moving body part. However, little is known about spatial limits on the effect of voluntary movements on simultaneous perception, especially when tactile stimuli are presented to a non-moving body part. We examined the effect of voluntary movement on the simultaneous perception of auditory and tactile stimuli presented to the non-moving body part. We considered the possible mechanism using a temporal order judgement task under three experimental conditions: voluntary movement, where participants voluntarily moved their right index finger and judged the temporal order of auditory and tactile stimuli presented to their non-moving left index finger; passive movement; and no movement. During voluntary movement, the auditory stimulus needed to be presented before the tactile stimulus so that they were perceived as occurring simultaneously. This subjective simultaneity differed significantly from the passive movement and no movement conditions. This finding indicates that the effect of voluntary movement on simultaneous perception of auditory and tactile stimuli extends to the non-moving body part.

We perceive the world using multimodal sensory information from the external environment. For instance, in watching a basketball match we usually see that the ball hits the ground and bounces and simultaneously hear the sound of the ball hitting the ground as the player is dribbling. Although light and sound originating from this event propagate through the air at different speeds, we perceive the visual and auditory information as a single event. That perception of visual and auditory information occurs simultaneously is surprising, given the lags in arrival and processing time of multimodal sensory information in the brain. This raises the question of how the simultaneous perception of multimodal sensory information is integrated in the brain to form a coherent representation of the world.

Such temporal perceptions of multimodal sensory information often accompany voluntary movements. It is known that voluntary movements can affect simultaneous perception^1^,^2^,^3^. Shi *et al*.^1^ reported that the point of subjective simultaneity (PSS) was 4 ms with the visual stimulus being first in the condition of voluntary finger movement combined with coherent visual feedback, whereas the PSS was 21 ms with the visual stimulus being first in no movement condition on a visual and tactile temporal order judgement (TOJ) task. The study demonstrated that voluntary movement with visual feedback significantly shifted the PSS of visual and tactile stimuli while voluntary movement without visual feedback did not induce significant difference. Additionally, Shi *et al*.^1^ reported that voluntary finger movement significantly decreased the just noticeable difference (JND), which is an indicator of temporal resolution. Several previous studies^2^,^4^,^5^ have reported divergent effects of voluntary movement on the simultaneous perception of auditory and tactile stimuli (i.e., PSS and JND), which might be attributable to the methods used. For instance, Kitagawa *et al*.^4^ and Nishi *et al*.^2^ found participants were able to predict the occurrence of the stimulus, and then decrease the JND, because a predictable stimulus can directly decrease the JND^6^,^7^,^8^. However, in Frissen *et al*.’s study^5^, the lack of short-range stimulus onset asynchronies (SOAs) might have concealed the difference between voluntary movement and no movement conditions. Recently, Hao *et al*.^3^ averted these putative effects of method procedures in previous studies, e.g., by eliminating the predictability of stimulus onset and using short-range SOAs, then replicated Nishi *et al*.’s^2^ finding that voluntary movement affected the PSS and Frissen *et al*.’s^5^ finding of no statistically significant difference in JNDs across the three conditions. Hao *et al*.’s finding^3^ also suggested that the improvement of temporal resolution in Nishi *et al*.’s study^2^ was caused by the predictability of stimulus onset, and the lack of difference in PSSs between voluntary movement and no movement conditions was concealed by the lack of short-range SOAs in Frissen *et al*.’s study^5^. Thus, these studies^1^,^2^,^3^ suggested that voluntary movements affected the simultaneous perception of visual–tactile stimuli and auditory–tactile stimuli, when the tactile stimulus was presented to the moving body part. In other words, the movement and tactile stimulus involved the same body part.

However, the same location of voluntary movement and tactile stimulus raises the question of whether voluntary movements affect the simultaneous perception of a non-moving body part. Until now, little has been known about spatial limits on the effect of voluntary movements on simultaneous perception, especially when tactile stimuli are presented to a non-moving body part.

The present study examined the effect of voluntary movement on simultaneous perception of auditory and tactile stimuli presented to a non-moving body part by a TOJ task, in which the order of auditory and tactile stimuli was judged. We chose the same conditions as in Hao *et al*.’s study^3^, including voluntary movement, passive movement, and no movement, to compare with the temporal effect of voluntary movement on a moving body part in Hao *et al*.’s study^3^. We also discussed the possible mechanism for this effect.

## Results

The TOJ task is often used to investigate the temporal perception of multimodal sensory information^9^,^10^,^11^,^12^,^13^,^14^,^15^, in which participants are asked to judge the temporal order of two stimuli presented at various SOAs. In the TOJ task, the PSS and the JND are used to measure simultaneous perception. The PSS^10^,^16^,^17^,^18^,^19^ is the point in time when two presented stimuli are perceived by an observer to occur simultaneously; the JND^9^,^10^,^20^,^21^,^22^ is the temporal resolution between the two stimuli.

We used three experimental conditions: voluntary movement, passive movement and no movement. In the voluntary movement condition, the participants were asked to voluntarily move their right index finger and judge the temporal order of auditory and tactile stimuli presented to their non-moving left index finger. In the passive movement and no movement conditions, the participant’s right index finger was moved by a device or held stationary, respectively, and participant judged the temporal order of auditory and tactile stimuli presented to the non-moving left index finger.

**Figure 1:**
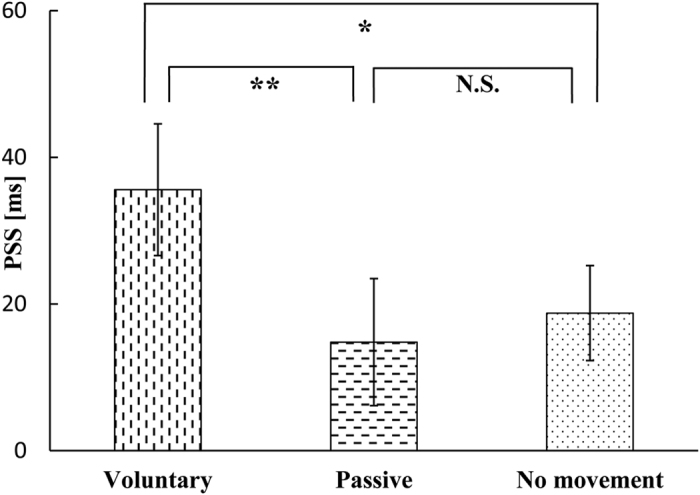
PSS results in the voluntary movement, passive movement and no movement conditions. On the abscissa are the three experimental conditions. On the ordinate is the PSS value. Error bars represent standard errors, *P < 0.05 and **P < 0.01.

The mean PSS (±standard error, SE) was 35.6 ± 9.0 ms for the voluntary movement condition, 14.8 ± 8.7 ms for the passive movement condition and 18.8 ± 6.5 ms for the no movement condition. A one-way repeated measures analyses of variance (ANOVA) with movement condition as a factor showed a significant effect (*F*(2, 34) = 7.60, P = 0.002). Subsequently, Bonferroni–Holm paired *t* tests revealed significant differences between the voluntary and passive movement conditions (P = 0.009) and between the voluntary and no movement conditions (P = 0.024). There was no significant difference between the passive and no movement conditions (P = 1.0), as shown in Fig. 1.

**Figure 2:**
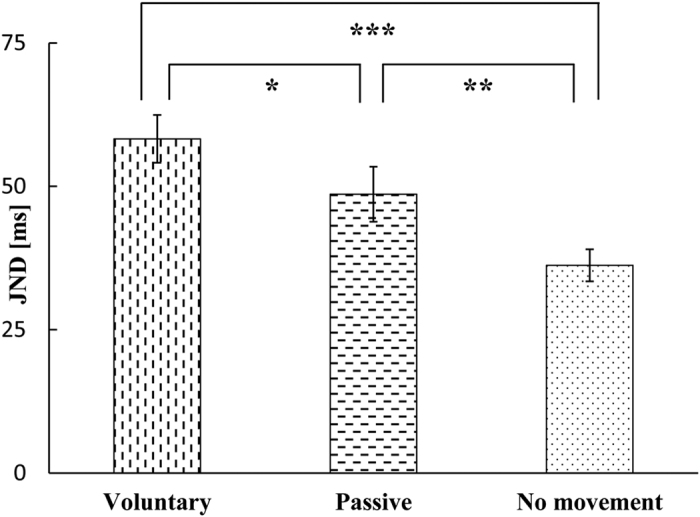
JND results in the voluntary movement, passive movement and no movement conditions. On the abscissa are the three experimental conditions. On the ordinate is the JND value. Error bars represent standard errors, *P < 0.05, **P < 0.01 and ***P < 0.001.

The mean JND (± SE) was 58.3 ± 4.2 ms for the voluntary movement condition, 48.6 ± 4.8 ms for the passive movement condition and 36.2 ± 2.8 ms for the no movement condition. A one-way repeated measures ANOVA with movement condition as a factor showed a significant effect (*F*(2, 34) = 19.87, P < 0.001). Subsequently, Bonferroni–Holm paired *t* tests revealed significant differences between the voluntary and no movement conditions (P < 0.001), between the voluntary and passive movement conditions (P = 0.036) and between the passive and no movement conditions (P = 0.005), as shown in Fig. 2.

## Discussion

The present study investigated whether the effect of voluntary movement on the simultaneous perception of auditory and tactile stimuli reaches a non-moving body part beyond the moving body part. Specifically, in the TOJ task, participants were asked to judge the temporal order of auditory and tactile stimuli presented to their non-moving left index finger, after they completed voluntary movement with their right index finger. We compared the PSSs and the JNDs in three experimental conditions: voluntary movement, passive movement and no movement. We introduced the passive movement condition, in which a device moved the participant’s body part, to remove the effect of proprioceptive sensation on simultaneous perception. We found that the voluntary movement significantly affected the PSS, compared with passive movement and no movement. Voluntary movement and passive movement significantly increased the JNDs, compared with no movement. These results indicated that voluntary movement also affected simultaneous perception of auditory and tactile stimuli, even when the tactile stimulus was presented to a non-moving body part, as well as to a moving body part as has been shown in previous studies^2^,^3^.

As shown in Fig. 1, there were significant differences in the PSSs between the voluntary and passive movement conditions, and between the voluntary and no movement conditions. Compared with the PSS in the no movement condition, the PSS in the voluntary movement condition, but not in the passive movement condition, significantly shifted (Table 1, Significance of Differences of PSSs row). This means that to be perceived simultaneously, the auditory stimulus needed to be presented before the tactile stimulus for a longer period in the voluntary movement condition than in the passive movement condition or no movement condition. Furthermore, there was no significant difference in the passive and no movement conditions. It seems that proprioceptive sensation in the movements did not affect the PSS. The above-mentioned statistical significance analysis of PSS results in the present study are consistent with those from our group’s previous study^3^, in which voluntary movement, compared with passive and no movements, was reported to affect the PSS of auditory and tactile stimuli for a moving body part. Thus, the results of both the present study and Hao *et al*.’s study^3^ indicated that rather than proprioceptive sensation in the movements, the voluntary movement affected the PSS (Table 1, Significance of Differences of PSSs row), when a tactile stimulus was presented to both the moving body part and the non-moving body part (Table 1, Location of tactile stimulus row).

As shown in Fig. 2, there were significant differences in the results of JNDs among the three conditions in the present study. In other words, the voluntary movement and passive movement, compared with the no movement, significantly impaired the temporal resolution (Table 1, Significance of Differences of JNDs row), and the impairment of temporal resolution by the voluntary movement was worse than the impairment of temporal resolution by the passive movement. While in Hao *et al*.’s study^3^, there was no significant difference of JNDs in the three conditions (Table 1, Significance of Differences of JNDs row), which means that the voluntary movement and passive movement, compared with no movement, did not affect the JND.

The impairment effects of the voluntary movement and passive movement, compared with the no movement, on the temporal resolution in the present study is inconsistent with that of Hao *et al*.’s study^3^ (Table 1, Significance of Differences of JNDs row). This inconsistency might be attributable to the different procedures in these two studies. In the present study, participants paid attention to the tactile stimulus on their left index finger on the TOJ task, and at the same time, the locations of the voluntary movement and passive movement were on their right index finger. In Hao *et al*.’s study^3^, participants paid attention to the tactile stimulus on the right index finger on the TOJ task, where the locations of voluntary and passive movements were. This means that in the present study, the attention, which should have been focused on the tactile stimulus on the left index finger, seems to be divided by the voluntary and passive movements of the right index finger. This impairment of temporal resolution by divided attention is consistent with the impairment of temporal resolution shown by Pérez *et al*.^24^, who suggested that divided attention might impair temporal resolution. Pérez *et al*.^24^ reported that JND increased in the TOJ task with two visual stimuli after they used an initial visual target stimulus to reduce the available attentional resource. Thus, the mechanism of impairments of temporal resolution by the voluntary movement and passive movement in the present study may be similar to those described in Pérez *et al*.^24^, although they used a visual target stimulus to divide attention and two visual stimuli in the TOJ task.

Here, the PSS shift in the voluntary movement condition might not be affected only by attention, as a prior entry effect in previous studies^4^,^25^,^26^,^27^,^28^,^29^. For example, in a visual and tactile TOJ task, there is a significant difference in the PSSs when participants are asked to pay attention to visual stimulus in one condition and to tactile stimulus in another condition. In the present study, the available attentional resource for the TOJ task seems to be decreased by the movements, especially by the voluntary movement. That is, the available attentional resource for the TOJ task in the voluntary movement condition may be smaller than in the passive and no movement conditions. The available attentional resource for tactile stimulus in the TOJ task may be smaller in the voluntary movement condition than in the passive and no movement conditions, because participants were asked to pay attention to tactile stimulus, rather than auditory stimulus. If this available attentional resource for tactile stimulus, rather than auditory stimulus, affects the PSS in the present study, as in a prior entry effect, the PSS in the voluntary movement condition may be smaller than the PSSs in the passive and no movement conditions. However, in the present study, the PSS in the voluntary movement condition was significantly larger than the PSSs in the passive and no movement conditions. Thus, only attention factor might not be enough to explain the results of PSSs in the three conditions. Additionally, this speculation might also occur in Hao *et al*.’s study^3^ as well since there was a significant PSS shift only in voluntary movement condition, but not in the passive and no movement conditions. Therefore, the PSS shift in the voluntary movement condition might not be affected only by attention in accordance with the prior entry effect as described in previous studies^4^,^25^,^26^,^27^,^28^,^29^, but might also be affected by a unique mechanism related to voluntary movement.

Efference copy, as a copy of the motor command^30^,^31^, generated in voluntary movement seems to be the ubiquitous factor to form a coherent representation of the external world during voluntary movements. Frissen *et al*.^5^ have suggested that the efference copy might affect the temporal resolution, though this effect occurred in the intramodal tactile stimulus pairs. Furthermore, efference copy is generated in the presupplementary motor cortex and the premotor cortex (PMC)^30^. PMC was reported to modulate the somatosensory cortex (S1), because the activations of PMC and S1 were found in a functional magnetic resonance imaging study, when participants were performing voluntary movements in the absence of proprioceptive feedback^32^. S1 and the secondary somatosensory cortex (S2) adjacent to S1 process inputs including touch^33^,^34^ from the various systems of the body. The S2 areas in the left and right hemispheres are densely interconnected, and stimulation on one side of the body will activate S2 areas in both hemispheres^35^. As mentioned above, the efference copy might be a possible reason for the effect of the voluntary movement on simultaneous perception. We speculate that the efference copy might not only predict the sensory feedback of movements^36^,^37^,^38^ and suppress self-generated sensory information^39^, but might also create unexpected side effects in the brain, such as potential effects on the integration of multimodal sensory information during voluntary movements.

In conclusion, our results indicate that voluntary movement affected simultaneous perception of auditory and tactile stimuli, even when the tactile stimuli were presented to a non-moving body part. Furthermore, we suggest that in the voluntary movement condition, the impairment of temporal resolution might be affected by decreased attention because of voluntary movement of the moving body part, whereas the PSS shift might be affected by attention as well as other mechanisms (e.g., efference copy) in voluntary movement.

## Methods

### Participants

Eighteen participants (2 females, 16 males; mean age: 24.2 years; range: 23–28 years) completed the experiment and were compensated for their participation. All participants were naïve to the purpose of the experiment. They were all right-handed and none exhibited any difficulty in moving their right index finger. All participants had a normal auditory threshold and sense of touch. Before administering the experiment, written informed consent was obtained from each participant. The Ethics Committee of the Tokyo Institute of Technology approved the study and the methods were carried out in accordance with their approved guidelines.

### Apparatus and stimuli

The tactile stimulus was an impulse force (3N, 10 ms, rectangular pulse) provided by a Geomagic^®^ Touch™ Haptic Device (Geomagic, Rock Hill, SC, USA). The stimulus was presented to each participant’s left index finger during movement or non-movement of the right index finger. Passive movement was provided by another Geomagic^®^ Touch™ Haptic Device. The auditory stimulus was a sinusoidal wave sound (2000 Hz, 50 dB, 10 ms) simultaneously presented to both ears using earphones (HP-RHF41, radius, Tokyo, Japan). The response machine was a triple foot switch (Strich Technology, Huizhou, China). The timing of the two presentations and the movements of the device were controlled to a margin of error of 1 ms. These sensory stimulation systems were operated by computer programmes installed on a PC workstation (Latitude E5430; DELL, Plano, TX, USA), developed with the Open Haptics software development toolkit (Geomagic) on the Microsoft Visual C++ 2008 platform (Microsoft, Redmond, WA, USA).

### Task and conditions

In the TOJ task, a pair of auditory–tactile stimuli was presented with varying SOAs (intervals between the auditory–tactile stimuli pair) and the temporal order of the two stimuli was judged by the participants. The SOAs were ±240, ±120, ±60, ±30 and 0 ms (in which the negative value indicates that the tactile stimulus was presented before the auditory stimulus, and vice versa). There were three experimental conditions: voluntary movement, passive movement and no movement.

### Procedure

**Figure 3:**
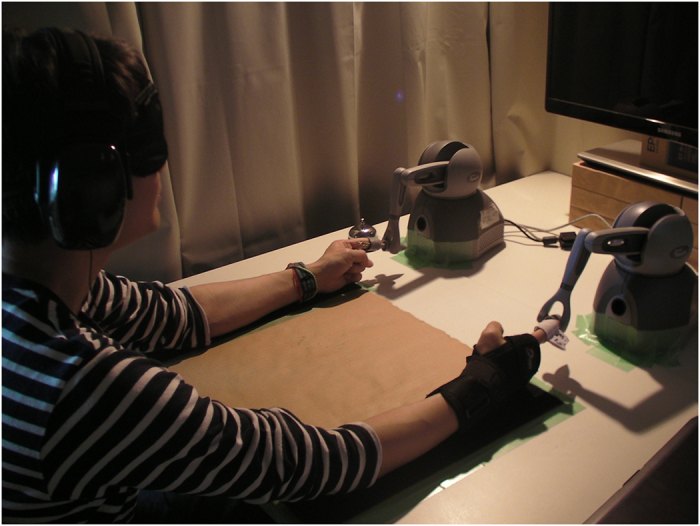
Experimental environment.

Participants were seated in a dark, sound-attenuated room in front of the stimulation systems with the palmar side of their right and left index fingers held in the haptic devices and the tactile stimulus on their left index finger. They also wore an eye mask to eliminate the confounding effects of visual stimuli during the experiment and sound-insulating ear muffs over the earphones (Fig. 3). Because both hands were engaged, participants were required to enter the temporal order of the auditory and tactile stimuli using a foot switch. The left key represented the presentation of tactile stimulus first and the right key represented the presentation of the auditory stimulus first. The mean rate of movement for the participants’ fingers was 75.73 mm/s (standard deviation, s.d. = 5.13) in the voluntary movement condition, and the mean rate of movement of the haptic device was 71.75 mm/s (s.d. = 1.55) in the passive movement condition.

**Figure 4:**
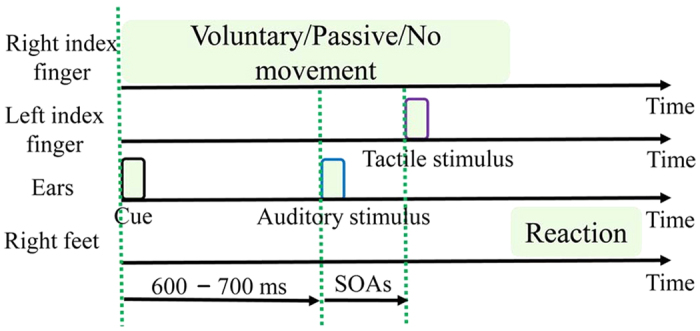
Schematic flow chart for one trial in each of the three conditions. “Voluntary/Passive/No movement” means voluntary movement, passive movement and no movement on the right index fingers in the voluntary, passive and no movement conditions, respectively. The interval between the cue and the TOJ task was randomly set from 600 to 700 ms. The durations of the cue, auditory stimulus and tactile stimulus were 10 ms. In the TOJ task, participants judged the temporal order of the auditory and tactile stimuli presented to the non-moving left index finger.

Voluntary movement condition: For each trial (Fig. 4), the participants began voluntarily moving their right index finger from right to left at their own pace. As they did so, a cue sound (distinct from the target auditory stimulus) indicated that the TOJ task was forthcoming. The first stimulus (either tactile or auditory) was then presented with a random delay of 600–700 ms after the cue sound onset. The second stimulus (auditory or tactile, whichever was not presented first) followed the first stimulus, offset by one of the nine SOAs previously mentioned. Here, the tactile stimulus was presented to the participants’ left index finger. Complete presentation of the two stimuli occurred during the voluntary movement. The participants then indicated which stimulus was presented first using a two-alternative forced-choice test to specify the temporal discrimination of the auditory–tactile stimuli pair. If the processing speed of the participants was not 50–110 mm/s, they were given one more trial, randomly chosen from the remaining trials.

Passive movement condition: Similar to the voluntary movement condition, one haptic device randomly started to move the participants’ right index finger for 500 to 1000 ms to reproduce the variance in the onset of voluntary movements in the preliminary experiments, while another haptic device presented the tactile stimulus to the participant’s left index finger. Complete presentation of the two stimuli occurred during the passive movement. The other procedures were the same as in the voluntary movement condition, including evaluation of the temporal order of the two stimuli and the SOA values. A speed of 76 mm/s for the finger movement was set for each trial (Fig. 4), which was considered a comfortable speed and representative of normal surface exploration. The movement trajectory of passive movement was reproduced by the movement trajectory of voluntary movements in the preliminary experiments.

No movement condition: The participants remained stationary throughout the no movement experiment with the palmar side of their left and right index fingers held in the haptic devices. The first stimulus (either tactile or auditory) was presented following a random delay (600–700 ms) after the cue sound onset (Fig. 4). The presentation of the second stimulus and the procedure for evaluating the temporal order of the two stimuli were the same as in the voluntary and passive movement conditions.

Each participant completed five blocks for all of the conditions in random order. Each block consisted of 45 trials; i.e., five trials for each SOA randomly selected from the following values: ±240, ±120, ±60, ±30 and 0 ms. The interval between trials was 1000 ms in each condition and white noise played in the background to effectively mask any sounds made by the haptic device. It took about five minutes for participants to complete one block of trials. They were given several minutes to rest between blocks, according to their preference. The order of the conditions was counterbalanced and the participants completed a total of 675 trials in the formal experiment; the entire procedure took about 3.5 hours across two successive days. During the experiment, the participants were asked to pay constant attention to the tactile stimulus to control for the prior entry effect^4^,^25^,^26^,^27^,^28^,^29^, which facilitates the processing of an attended stimulus compared with an unattended stimulus.

In the practice sessions, participants were asked to close their eyes and judge the order of the two stimuli and then open their eyes to see the feedback on the computer screen for each trial. With no information about the forthcoming condition, they first completed 90 trials of the no movement condition, then 45 trials each of counterbalanced voluntary movement and passive movement conditions. The SOA during the practice session was randomly chosen from ±240, ±120, or ±60 ms. In addition, participants each completed a practice run of 10 trials in which only the tactile stimulus was presented so they became accustomed to the appropriate finger speeds in the voluntary movement condition. They were given 2–3 minutes of rest before each block of trials in the voluntary movement condition to eliminate any potential practice effect.

## Data analysis

**Figure 5:**
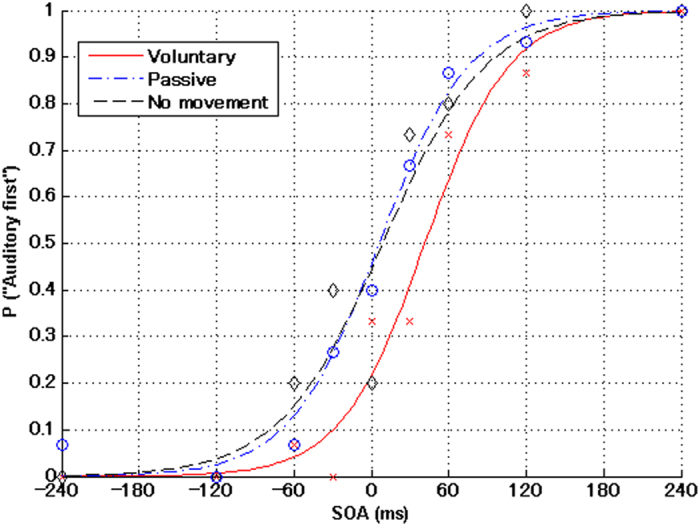
Average psychometric functions between all blocks in the voluntary movement, passive movement and no movement conditions for one participant. On the abscissa are the SOAs. On the ordinate is the proportion of times that the auditory stimulus was perceived before the tactile stimulus. Negative SOA value means that the tactile stimulus was presented before the auditory stimulus, and vice versa. The curves were estimated using the generalized linear model (see text for details). Voluntary represents the voluntary movement condition; Passive represents the passive movement condition; and No movement represents the no movement condition.

We used MATLAB Statistics Toolbox (MathWorks, Natick, MA, USA) for the statistical regression calculations and graphic representation of the results. First, we calculated the ratio of the answers for each SOA, in which the auditory stimulus was perceived first. Then we conducted the logistic regressions using a generalized linear model with the ratio from each condition. We fitted psychometric curves to the distribution of the mean TOJ for the voluntary movement, passive movement and no movement conditions, as shown in Fig. 5. The PSS and JND values were calculated for each participant in the regression analysis, based on three equations^40^:


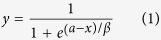



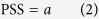



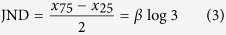


Here, *α* represents the estimated PSS, *x* denotes SOA, *β* is related to the JND and *x*_*p*_ represents the SOA with *p* as the percent of “auditory first” responses. Next, statistical analyses of the data were conducted to obtain the mean and standard error values for each condition.

## Additional Information

**How to cite this article**: Hao, Q. *et al*. Voluntary movement affects simultaneous perception of auditory and tactile stimuli presented to a non-moving body part. *Sci. Rep*. **6**, 33336; doi: 10.1038/srep33336 (2016).

## Figures and Tables

**Table 1:** Comparison between the present study and Hao *et al*.’s study^3^.

		Present study			Hao *et al*.’s study		
Location of tactile stimulus		Non-moving body part			Moving body part		
Differences of PSSs	Conditions	V–P	V–N	P–N	V–P	V–N	P–N
	Significance	**	*	N.S.	**	**	N.S.
	Effect size	0.81	0.71	0.17	0.84	1.04	0.29
	95% CI	0.11	0.01	–0.5	0.13	0.31	–0.39
		1.52	1.41	0.85	1.54	1.76	0.97
Differences of JNDs	Conditions	V–P	V–N	P–N	V–P	V–N	P–N
	Significance	*	***	**	N.S.	N.S.	N.S.
	Effect size	0.66	1.39	0.88	0.43	0.41	0.03
	95% CI	–0.03	0.63	0.17	–0.26	–0.28	–0.64
		1.36	2.14	1.59	1.11	1.09	0.71

V = voluntary movement condition, P = passive movement condition, N = no movement condition.

V–P, V–N, and P–N indicate the differences between the respective conditions. *P < 0.05, **P < 0.01, ***P < 0.001. N.S. = no significant difference. Effect size was calculated by Cohen’s d (d = 0.2, a small effect, d = 0.5, a medium effect, and d = 0.8, a large effect)^23^, 95% CI = 95% confidence interval of Cohen’s d.
